# Synergy Effects in the Chemical Synthesis and Extensions of Multicomponent Reactions (MCRs)—The Low Energy Way to Ultra-Short Syntheses of Tailor-Made Molecules

**DOI:** 10.3390/molecules22030349

**Published:** 2017-02-25

**Authors:** Heiner Eckert

**Affiliations:** Department Chemie, Technische Universität München, Lichtenbergstr. 4, Garching 85747, Germany; eckert@tum.de; Tel.: +49-89-354-5532

**Keywords:** multicomponents, MCR, post condensation modification/cyclisation PCM/PCC, MFCR, multifunction catalysis, variability, diversity, complexity, efficiency of synthesis

## Abstract

Several novel methods, catalysts and reagents have been developed to improve organic synthesis. Synergistic effects between reactions, reagents and catalysts can lead to minor heats of reaction and occur as an inherent result of multicomponent reactions (MCRs) and their extensions. They enable syntheses to be performed at a low energy level and the number of synthesis steps to be drastically reduced in comparison with ‘classical’ two-component reactions, fulfilling the rules of *Green Chemistry*. The very high potential for variability, diversity and complexity of MCRs additionally generates an extremely diverse range of products, thus bringing us closer to the aim of being able to produce tailor-made *and* extremely low-cost materials, drugs and compound libraries.

## 1. Prologue

**Figure 1:**
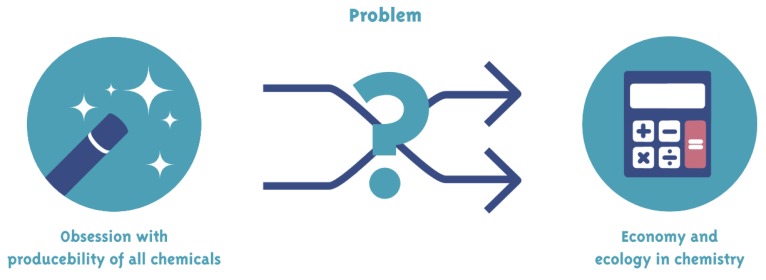
Problem in chemical syntheses relating to terms of producibility, ecology and economy.

**Figure 2:**
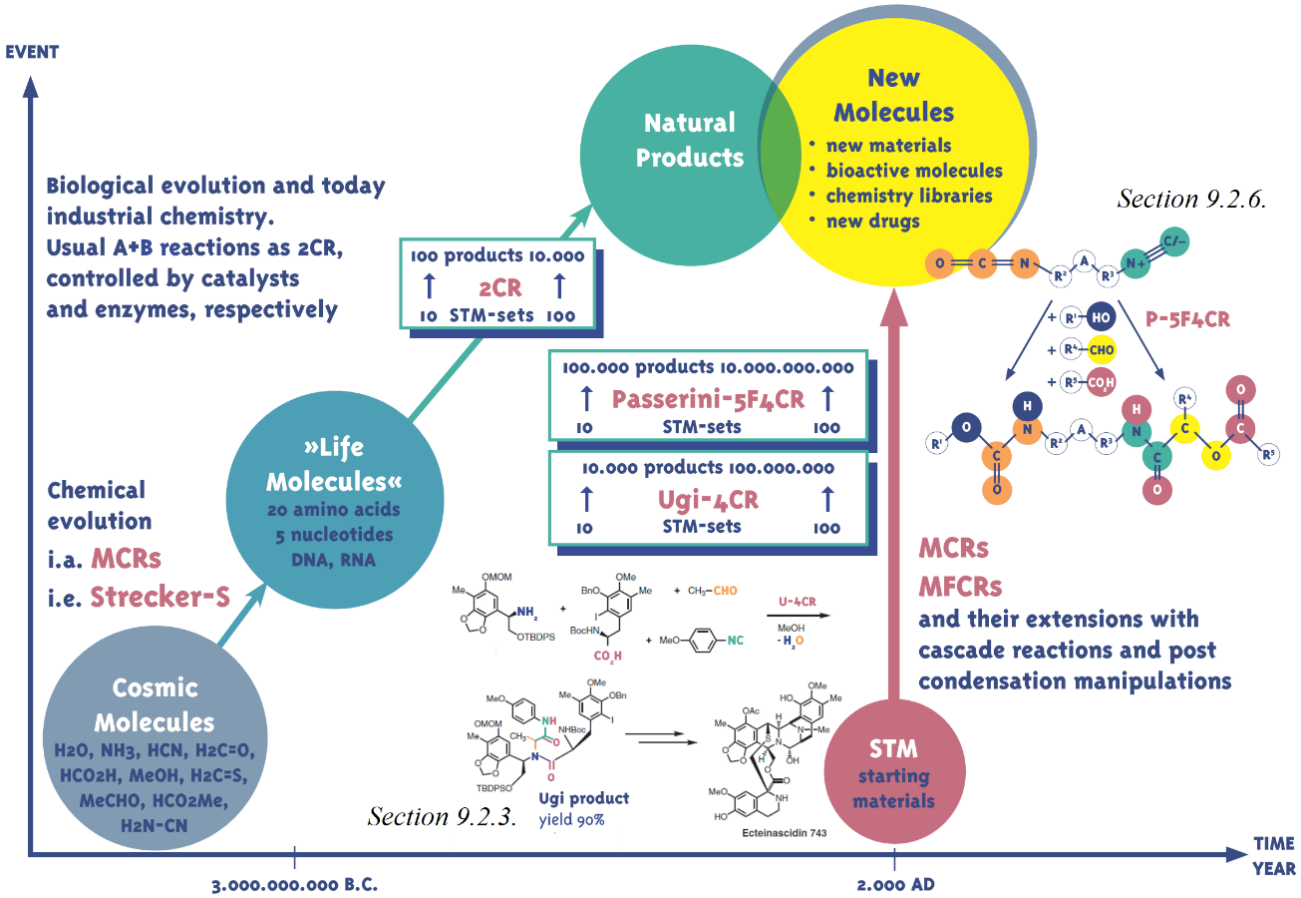
Significant systemic differences in syntheses of chemical products by means of two- and multicomponent reactions, respectively.

The great discrepancy between the traditional feasibility mania and rational efficiency assessment in chemical synthesis became immediately apparent to the author while working on his thesis and during everyday laboratory life. On the one hand, classic peptide synthesis with an inefficient, extremely high number of synthesis steps and on the other hand, elegant multicomponent reactions that can make such efforts unnecessary. This double strategy in chemical synthesis has given the author cause to reflect (Figure 1) on how to close this gap using a consistent procedure. Thereby significant systemic differences in syntheses of chemical products play an essential role (Figure 2).

## 2. Introduction

Special events in the author’s Vita runs synchronously alongside the scientific text and are indicated by alphabetically-ordered notes in Section 13. The scientific work carried out by the author spans a wide range and encompasses the development of new methods and reagents, as well as the defusing of hazardous substances. These methods and reagents flow into the section on syntheses by MCRs. Their applications in various areas of chemical synthesis include:

*Tools, novel methods, catalysts and reagents to improve organic synthesis*

Vitamin B_12_ models. Structures, supernucleophilicity.
Protective group techniques. Peptides such as β-lactam antibiotics, *N*-heterocyclic compounds and natural substances, such as camptothecin, using novel protective group techniques (2,2,2-trichloro-*tert*-butyloxycarbonyl (TCBOC) residue, cobalt-phthalocyanine).
Metal phthalocyanines. Poisoning-resistant hydrogenation catalysts, palladium-phthalocyanine with three switchable, partially orthogonal catalysis patterns, alamethicine sequence.
Peptide chemistry. Peptides such as human-β-endorphin sequences, Leu-enkephalin, polyamino acids and cyclopeptides, using the novel ferrocenylmethyl masking group.
Triphosgene. Safe syntheses with triphosgene and their up-scaling processes.
Synthesis efficiency. Quantitative assessment of a synthesis.

*Concerted simplifying synthesis chemistry on MCR basis*

Multicomponent reactions (MCRs). Systemic and post-MCR extensions.
MCR synthesis efficiency. Special preconditions. Algorithm on efficiency.
MCRs’ future. Concerted simplification of chemical processes.

The individual sections are seamlessly meshed, and thus methodologically produce what are to some extent substantial synergistic effects. All these different advantageous methods and reagents listed in Section 3, Section 4, Section 5, Section 6, Section 7 and Section 8 are developed *tools* for efficient syntheses. They support multicomponent reactions in particular, Section 9, Section 10 and Section 11 through their systemic property which strongly reduces the number of synthesis steps and promoting simplification of synthesis chemistry. Nearly all referred literature in Section 9 and Section 11 are reviews about the relative domain.

## 3. Vitamin B_12_ Models

[A] The idea was to find a cobalt(I) complex [1] that demonstrated the reactive chemical properties of vitamin B_12_ and cobaloximes [2], as well as constituting a stable reagent or catalyst. The choice fell on the light- and color-fast pigment cobalt-phthalocyanin, CoPc, **1** [3] [B]. Its properties were then to be investigated, in particular the nucleophilicity of its anion [PcCo^I^]^−^ **1a** in the oxidation stage +1 of the metal [4].

The nucleophilicity of [PcCo^I^]^−^ was determined conductometrically [1]. Table 1 shows the comparison with standard strongly nucleophilic agents [5], vitamin B_12s_ and its model cobaloxime(I), whereby **1a**, together with the latter, has a value >10 and is therefore designated a supernucleophile.

## 4. Protective Group Techniques

**Scheme 1:**
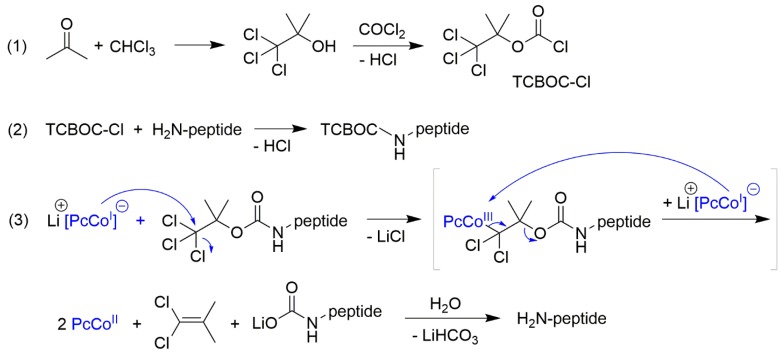
Introduction of TCBOC-residue (1) and its cleavage by means of **1b** (3).

Suitable protective groups play a central role in classic chemical peptide synthesis. Although the permanent protection of α-amino and carboxyl functions is well provided for by Z, BOC, ALOC, FMOC, TMS, benzyl and *tert*-butyl groups, there is still a need for selective orthogonal intermediary protective groups for the protection of amino acid third functions or special applications. A useful solution is provided here by using β-halogenalkyl and β-halogenalkoxy-carbonyl groups [6], which can be cleaved off by reduction in a weakly acidic environment, e.g., using zinc. The ideal protective group is one which is stable in acids and alkaline solutions, and not affected by catalytic hydrogenolysis, and which can be cleaved off under conditions that do not attack most of the other protective groups (orthogonality). This method was developed [7,8,9] and optimized [10,11,12,13] by Eckert and Ugi using selective reagents following the defined mechanism of reductive fragmentation as per Scheme 1.

**Figure 3:**
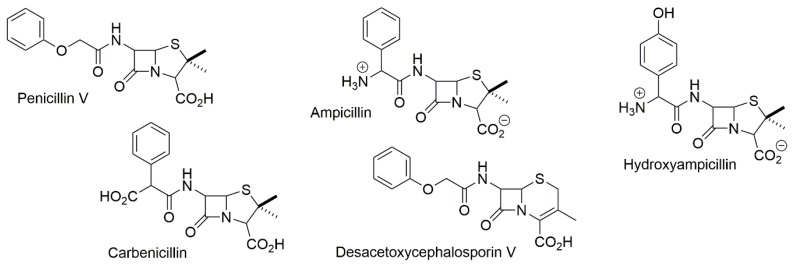
Unstable β-lactam antibiotics synthesized using protective group technique of Scheme 1.

The lithium salt of Cobalt(I)-phthalocyanin, Li[Co^I^Pc] **1b** (Section 3), was the selected reagent which met all conditions: fast reaction at room temperature (RT) in neutral environments, no reaction with other functional groups (except for the nitro group), high product yields and complete regeneration capability of the fully stable reagent. The cleavage of the protective groups can also be carried out catalytically on Co^II^Pc **1** with NaBH_4_ [12]. As an optimal protective group, the acid and base-stable 2,2,2-trichloro-*tert*-butyloxycarbonyl-residue (TCBOC) was created, which is introduced via its stable chloride TCBOC-Cl and can be cleaved off in just 1 min using **1a** (Scheme 1) [10]. Due to the extremely mild conditions, the method has been successfully used for the semi-synthesis of unstable β-lactam antibiotics, such as penicillin and cephalosporin derivatives (Figure 3) [13,14,15,16] [C].

## 5. Metal-Phthalocyanines, Hydrogenation Catalysts, Palladium-Phthalocyanin

### 5.1. Cobalt-Phthalocyanine as Reagent

**Scheme 2:**
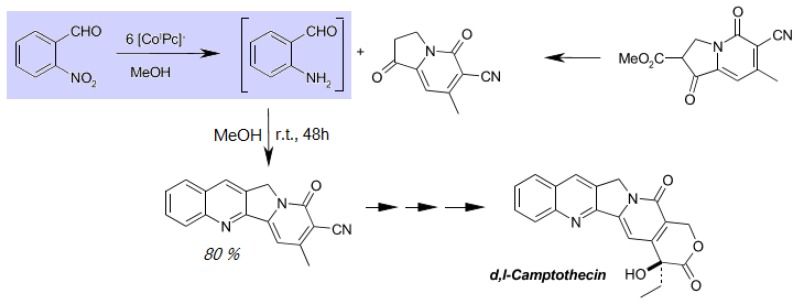
Camptothecin synthesis using **1a** in a Friedländer synthesis forming *o*-amino-benzaldehyde.

The reagent Li[Co^I^Pc] stands out due to its almost unique selectivity: apart from the reductive fragmentation, practically only the nitro group is reduced to the primary amine function [17,18]. All other normal functional groups remain intact, which makes **1a** particularly suitable for the in-situ preparation of aromatic o-amino-aldehydes in the Friedländer synthesis reaction. A significant improvement in the total synthesis of the frequently used anticancer drug camptothecin could be achieved (Scheme 2), whereby the yield of step 6 was increased from 20% to 62% [18] and up to 80%.

### 5.2. Hydrogenation Catalysts

In order to determine the capabilities of the vitamin B_12_-related Co^II^Pc and analogous metal-phthalocyanines (MPc), the reactive and the reductive properties of MPc in particular were researched with the metals M = VO, Mn, Fe, Co and Pd as catalysts using the reduction agents hydrogen and sodium borohydride [19,20]. The pronounced poisoning resistance of Co^II^Pc against strong catalyst poisons such as I^**−**^, CN^**−**^ or R-S^**−**^ was impressive.

### 5.3. Palladium-Phthalocyanine [D]

**Scheme 3:**
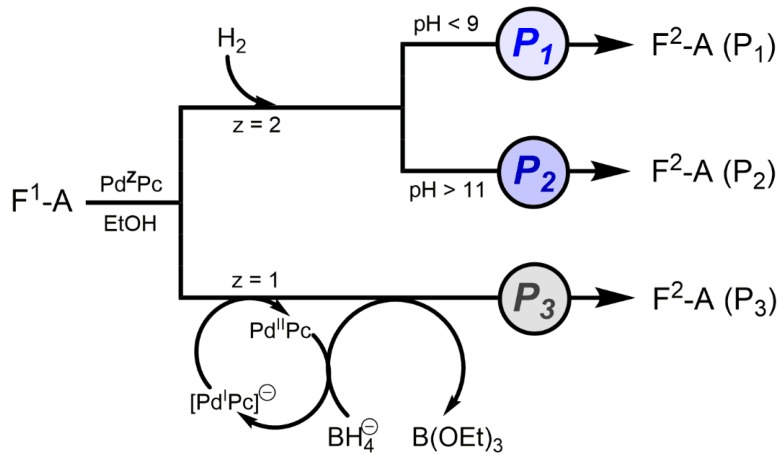
Pd^z^Pc as hydrogenation catalyst with 3 switchable catalysis patterns P_1_–P_3_ (Table 2) dependent on valence **z** in Pd^z^ and basicity of reaction medium (Figure 4).

**Figure 4:**
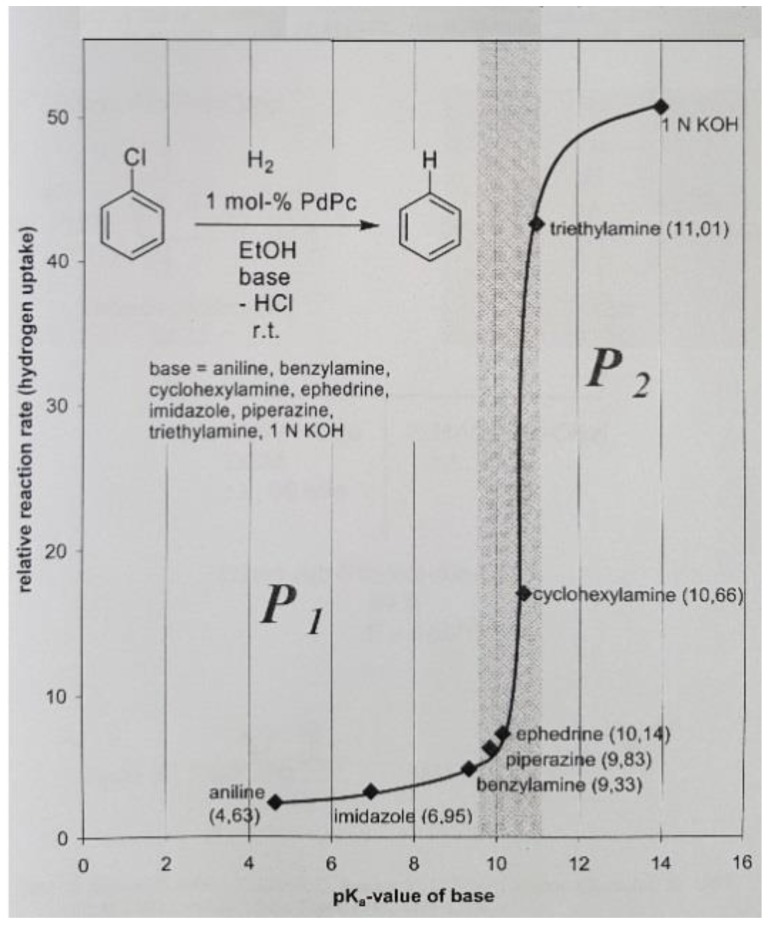
Graph of the sharp crossover from P_1_ to P_2_ caused by the basicity of reaction medium.

**Scheme 4:**
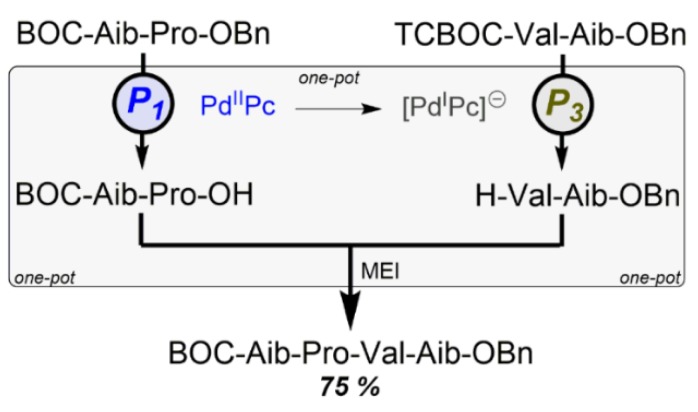
Reaction control by catalyst Pd^**z**^Pc in the one-pot synthesis of the alamethicin [13-16] sequence.

A prominent role is played here by palladium-phthalocyanin **2**, which can act with three different switchable specificities as Pd^II^Pc **2** and Na[Pd^I^Pc] **2a** as well as **2a** in alkaline environments (pH > 11, Figure 4), thereby generating three discrete catalyst patterns P_1_–P_3_ (Table 2 and Scheme 3) [20,21]. These are also partially orthogonal to each other and thus open up interesting new options for synthesis. This “catalytic doubling” has been successfully applied in a one-reactor reaction during the synthesis of the partial sequence [13-16], BOC-Aib-Pro-Val-Aib-OBz, of the peptide antibiotic alamethicin (Scheme 4).

The starting substance was the protected dipeptide BOC-Aib-Pro-OBz. After P_1_, the benzyl residue is selectively cleaved off with H_2_ at **2**. After adding of TCBOC-Val-Aib-OBn the TCBOC residue can be removed with inverse selectivity after P_3_ at **2a** through the addition of NaBH_4_. This leaves the benzyl residue intact. The final coupling using morpholinoethyl isocyanide delivers the protected tetrapeptide with a yield of 75% via three steps in one reactor (Scheme 4). Synthesis efficiency (for this item see Section 8) is Eff_Synth_ = 75%, whereas that of the three single reactions with very good yields in [21] is Eff_Synth_ = 23% only. The **2**/H_2_/EtOH system is air-stable and the catalyst Pd^II^Pc can be quantitatively recovered and re-used without further treatment.

This means the catalyst **2**/**2a** has an unusually high *synergistic* potential: using the same catalyst means that orthogonal reactions in a one-reactor method can proceed without prior isolation and preparation, thus *saving* two out of three practical synthesis steps! Therefore, the application of protective group techniques may no longer be an efficiency brake in syntheses; e.g., this method could help make the eurystatin synthesis in Section 9.2.4 even more efficient by saving up to three steps.

## 6. Peptide Chemistry—The Ferrocenylmethyl (Fem) Masking Group

**Figure 5:**
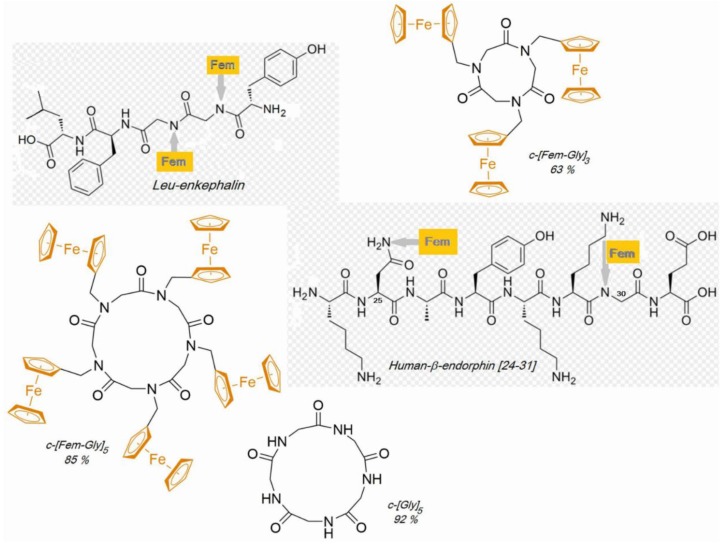
Synthesis of peptides otherwise hardly accessible, via their Fem-derivatives.

Peptide synthesis is often faced with the problem of difficult or practical insolubility of sequences in solvents if corresponding conformations of the sequences can be taken. Preventing this requires reversibly masking the peptide bonds. This is most favorably achieved using the ferrocenylmethyl (Fem) residue [22], which can simply be introduced via the easily accessible ferrocenaldehyde on the amino function of the respective amino acid in a reductive alkylation reaction with H_2_ at Pd^II^Pc and removed with trifluoroacetic acid in dichloromethane. Various human-β-endorphin sequences [23], Leu-enkephalin and hexaglycine [24] and the cyclopeptides *cyclo*-triglycine and *cyclo*-pentaglycine [25] could be produced with this method under mild conditions with far better yields than previously possible (Figure 5). Also of interest is the further useful application of Pd^II^Pc in selective reductive alkylation. The Fem residue transfers modified properties here to the peptides:

- Lipophilisation of the peptide bonds. The Fem peptides mentioned above dissolve well in standard non-polar solvents such as ethyl acetate and ethyl acetate/hexane mixtures, even to some extent in pure hexane! This effect is particularly favorable on peptide bonds of glycine [23,24,25].
- Strong chromophore. Due to their high lipophility, Fem peptides can be easily and inexpensively purified using silica gel chromatography and ethyl acetate/hexane mixtures. Implementation is facilitated by the strong intrinsic orange colour of all Fem derivatives.
- Electrochemical Detection (ECD). Detection following chromatography can also be done with ECD. The detection limit here is very low with 10^−15^ M (femto-molar!) [26]. The Fe^II^ in the ferrocene of the Fem residue is the electrophore.
- Conformational effects. Strong steric hindrance and high lipophilicity of the Fem residue have a significant influence on the conformational effects of the Fem peptide.

During Leu-Enkephalin synthesis, masking of both peptide bonds on the Gly-Gly sequence prevents the formation of a side product that would occur more substantially without Fem masking [24]. In *cyclo*-triglycin synthesis, the strong effect of all-Fem masking is particularly evident: under mild reaction conditions (6 h at RT), the output is 63% *cyclo*-(Fem-Gly)_3_ from H-(Fem-Gly)_3_-OH. *Cyclo*-(FemGly)_5_ from H-(FemGly)_5_-OH runs with 85% yield, what from the free *cyclo*-(Gly)_5_ is obtained with 92% yield [25] (Figure 5). Also, a severe solubility problem during the synthesis of the octapeptide [24-31] sequence of human-β-endorphine has been solved successfully by using Fem-residues at [30] Gly and [25] Asn-positions (Figure 5) [23].

## 7. Triphosgene—A Safe Phosgene Substitute

[E] The elemental analysis of triphosgene suggested a molecular formula of COCl_2_, which was baffling and gave rise to the conclusion that this solid could be a solid phosgene. An IR spectrum provided information about the composition: it was not the cyclic trimeric hexachlor-1,3,5-trioxane, but rather bis(trichloromethyl)carbonate **3**. The ^13^C-NMR spectrum with two signals confirmed this structure.

**Scheme 5:**
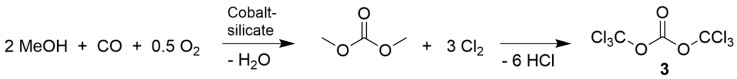
Triphosgene synthesis from dimethylcarbonate and chlorine.

Synthesis of **3** was rather simple: radical chlorination of dimethyl carbonate (DMC, Scheme 5). The up-scaling of the reaction to a 20-L flask resulted in a yield per batch of approx. 20 kg (99%) triphosgene with a melting point of 80 °C [F]. DMC is a million-ton product that is industrially produced using methanol, CO and O_2_ (Scheme 5). Compound **3** could replace all actual reactions of phosgene [27,28,29], was therefore named as triphosgene and as a crystalline solid it is significantly safer to handle than the gaseous phosgene **4**, as can be seen in Table 3.

Triphosgene is preferably used to synthesize isocyanides applied in the production of important isocyanide-based IMCRs [27,28,29], as described in Section 9.2.

**Scheme 6:**
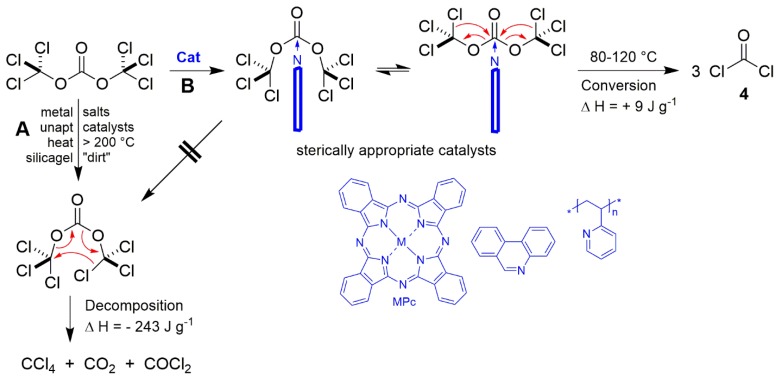
The two pathways of triphosgene transformation [29,30].

Contrary to most opinions, phosgene is not just produced during the thermal decomposition of triphosgene, instead there are two defined ways of transformation as per Scheme 6, whereby the thermal decomposition to phosgene, carbon dioxide and tetrachloromethane (pathway **A**) always occurs around approx. 200 °C and catalyses according to a 6-element mechanism, because as is thermodynamically determined, ΔH = −243 J·g^−1^. If the transformation is enabled at relatively lower temperatures of 80–120 °C, pure phosgene (pathway **B**) is output in a reconversion without reaction heat, ΔH = +9 J·g^−1^, if an appropriate catalyst is used that also blocks the 6-element mechanism [30].

There are reactions and processes in synthesis chemistry that run better with phosgene than with triphosgene, as i.e., the production of high-chem isocyanato-isocyanides for MCR chemistry in Section 9.2.6 [28,29,30]. This process was easily up-scaled to the 5 kg range and a phosgene generator was designed for a 30 kg·h^−1^ throughput [29]. Thus, also the supply-chain of phosgene in bulk quantities becomes safer: triphosgene for transport and storage, phosgene as reagent. As a highly remarkable instance, MPc are also absolutely stable and re-usable catalysts for this process [G].

## 8. Synthesis Efficiency

The smallest unit, the cell, of chemical synthesis is the (synthesis) step. Its evaluation criteria are:

- A logical synthesis plan with environmentally friendly processes and starting materials (STM), the least possible side products (additional reactions),
- access to and price of starting materials,
- cost of implementation and purifying the product and
- foremost, the *yield*.

This is the sum criterion with a high intrinsic chemical component and the yardstick for every synthesis step. Its significance is well known by every chemist. The observer will drastically notice the relation between the overall yield y_oa_ and the number of steps **n** as clearly shown in Table 4: To facilitate understanding, intermediate yields y_av_ are applied on a case by case basis.

The number **n** of all product-generating steps is the second main criterion for assessing a synthesis. “The less, the better” is the motto here and most chemists do not realize how great the influence of the number of steps is on synthesis efficiency. As yield and step number are primarily factual criteria (mol or quantity), they provide a sound basis for evaluating a synthesis. In combination, they are a reliable criterion for the quantitative overall evaluation of a synthesis, i.e., its efficiency.

**Scheme 7:**
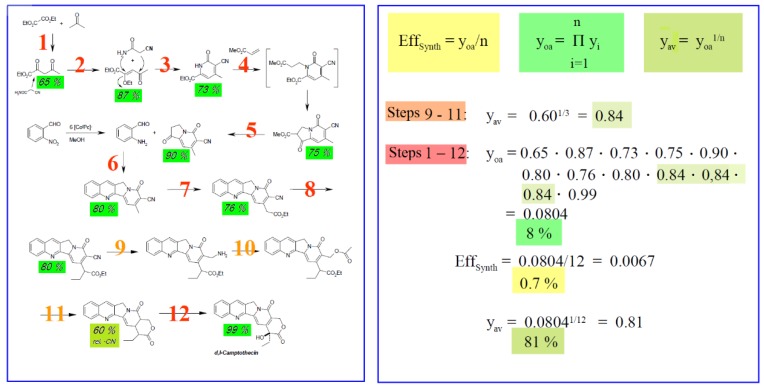
Efficiency of synthesis Eff_Synth_ of the camptothecin synthesis in Section 5.1 Scheme 2. y_i_: yield of one step; n: number of steps; y_oa_: over-all-yield over n steps; y_av_: geometric average yield over n steps; Eff_Synth_: efficiency of synthesis.

The synthesis efficiency Eff_Synth_ (Scheme 7) follows from the overall yield **y_oa_** and number of steps n of a synthesis, where the overall product yield equals the product of all the individual yields per step. A value which better reflects the yield is the average step yield y_av_. Scheme 7 represents the data for the camptothecin synthesis in Section 5.1 Scheme 2 [18] on the basis of these evaluation criteria.

**Scheme 8:**
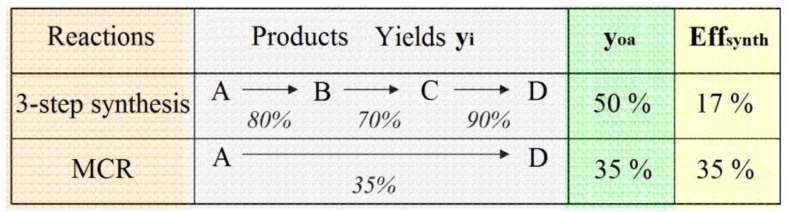
Case study of two ways of synthesis to form product D.

Syntheses of a product D with various numbers of steps can vary greatly in efficiency, as shown in Scheme 8. Even when the yield of the MCR is only moderate, its synthesis efficiency still is twice as high as that of a 3-step synthesis with its good yield. The synthesis efficiency tool helps to assess and forecast synthesis schemes. Section 10 concerns the development of an algorithm relating to the efficiency of MCRs with regards to parallel reactions.

## 9. Multicomponent Reactions (MCRs)

The prominent role of Multi-Component Reactions (MCRs) in the chemical evolution of the range of cosmic molecules (Figure 2) to the molecules of life, such as amino acids and nucleobases, became clear as the selecting agents, the enzymes, were not yet available at that time. Biological evolution over billions of years consequently selected the pathway of the enzyme-controlled, highly selective two-component reactions. The domain of chemical synthesis that expanded exponentially over the past 200 years retained this scheme even though, since the middle of the 19th century, extremely important MCRs such as the Strecker and Hantzsch dihydropyridine syntheses, and the Biginelli reactions represented a yet small, but profoundly important part of the wealth of chemical reactions. Only the isocyanide-based Passerini and Ugi reactions (IMCRs) in the 20th century [31] gave access to a versatile and broad range of prospective syntheses. MCR chemistry has seen a very fast upswing (over 500 reviews) since the turn of the millennium, while the number of new MCRs and their application is increasing steadily, demonstrated in some reviews [32,33,34,35,36,37,38,39,40,41].

**Scheme 9:**
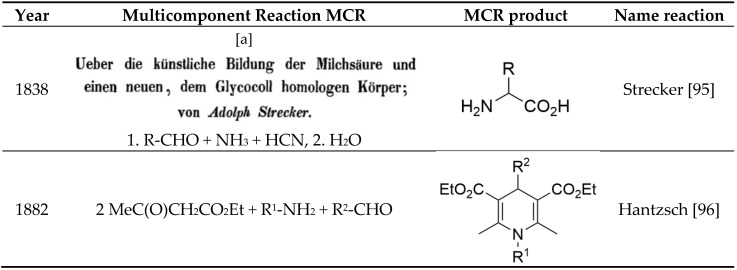

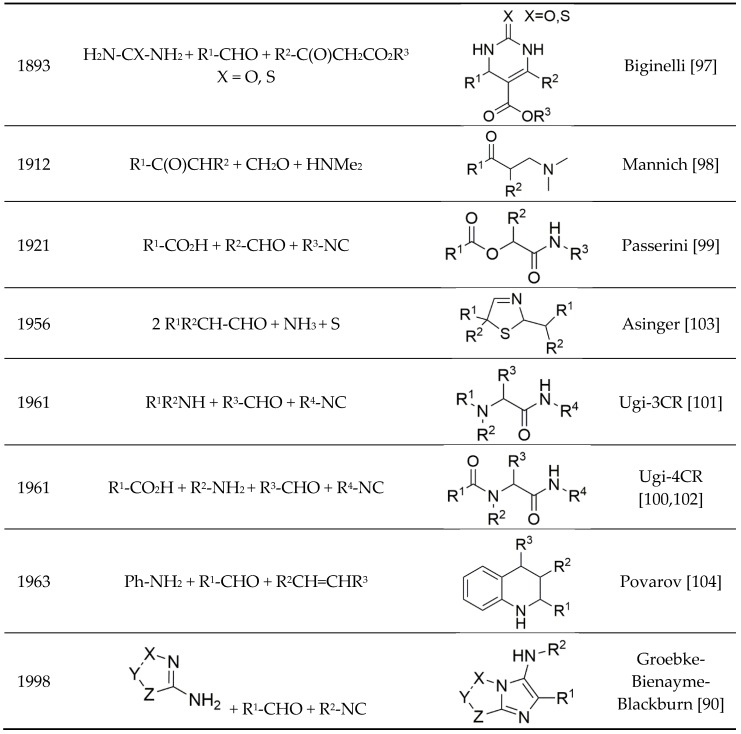
Well-known multicomponent reactions (MCRs). [a] On the Artificial Formation of Lactic Acid and a New Compound Homologous to Glycine; by Adolph Strecker.

IMCRs [38,42,43,44,45,46,47,48,49,50], alkyne-based [38,51,52,53,54] and C-H-acidic compound [55,56,57,58,59,60]-based MCRs in particular have a high potential to be very diverse. Many novel MCRs [57,58,59,60,61,62,63,64,65,66,67,68,69,70,71,72,73] have meanwhile become part of the range of syntheses, e.g., boron-mediated [62,66], photo-induced [67] and carbene-based MCRs [72] such as N-heterocyclic carbenes (NHCs). They all increase the application options for MCRs, such as MCR nanosystems and mechanochemical reactions [65]. MCRs have been also applied in polymer [74], nucleoside [75] and carbohydrate [76] chemistry. Main field of application remains the production of heterocyclic compounds and many natural products by means of MCR [38,42,77,78,79,80,81,82,83,84,85,86,87,88], leading directly towards drug design and discovery [89,90,91,92,93]. Scheme 9 presents a current brief of well-known multicomponent reactions [35,40,55,90,94,95,96,97,98,99,100,101,102,103,104,105,106,107,108].

### 9.1. Definition of MCR, Extensions and Optimal Reaction Conditions

Formally, every reaction with three or more reaction partners is a multicomponent reaction. The difference from 1CR and 2CR is easily understood, while the overall chemical reactions almost completely occur in the latter. Nature chose this pathway at a very early stage of evolution (Figure 2). All MCRs are characterised by the integration of all reaction partners (components **C**) in just *one* reaction product and, to put it succinctly, thus generate addition reactions which inherently do not show by-products. MCRs do not easily fit into the conventional way of thinking. Small molecules such as H_2_O can for example be released in the MCR. An exact definition for this effect still does not exist today. MCR mechanisms have been partly clarified but cannot be easily reproduced [35,109]. Statistical models for the kinetics fail because while third or higher grade collisions are highly improbable, MCRs nevertheless run smoothly and rapidly. The preferred interactions of the individual components with each other are often not yet fully understood but do deliver overall very functional reactions such as the Gewald reaction. The thermodynamic assessment of the MCRs [110,111] generally results in a “temperate balance” (cf. Bach’s “Well-Tempered Clavier”) where the reaction energies are slightly negative, reactions are self-propelled and do not require intensive thermal control. The atomic balance of the MCRs is usually excellent because all molecular parts remain present in the target molecule as structural elements. Variability, diversity and complexity of the target molecules are high [112,113] and can be controlled via the arrangement and nature of the MCRs. The distinct capacity for one-reactor syntheses also render MCRs fit for large-volume productions.

**Scheme 10:**
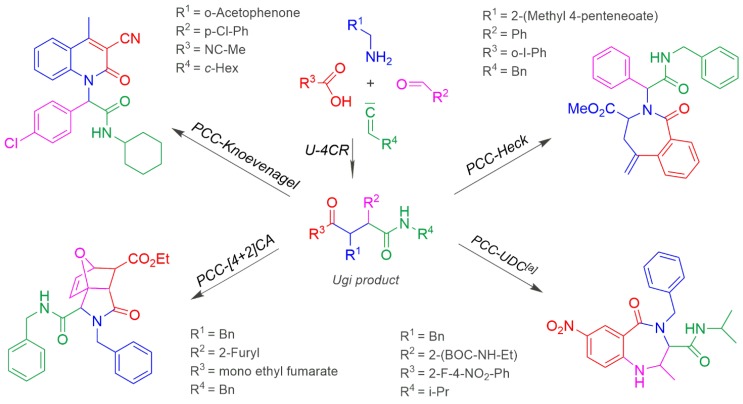
Post-condensation cyclisations (PCCs) of an Ugi-reaction. [a] UDC: Ugi deprotection cyclization. BOC (*tert*-butoxycarbonyl) protective group is cleaved by trifluoracetic acid.

The efficiency of MCRs can be increased by extensions without adding additional reaction partners, because functional groups are present in the STM that react in Domino-reactions with the newly created functional groups in the MCR as post-condensation modification (PCM) and post-condensation cyclisation (PCC) [87,112,113,114,115,116,117,118,119,120,121,122]. Scheme 10 shows examples of several Tandem-U-4CR/Diels-Alder, Knoevenagel, Heck or UDC reactions as PCCs [87].

Suitable solvents for MCRs are alcohols such as methanol and ethanol, water [123,124,125] and ionic liquids (ILs) [126,127,128,129,130,131], PEG-based ILs [132]. Unconventional solvents [133] such as trifluoroethanol [134] were also investigated. Solvent-free MCRs [135,136] also provide good results.

Even though MCRs normally run without catalysts, there are special applications for both various transition metal catalysts [79,116,137,138,139,140,141,142,143,144] and organic catalysts [79,145,146,147,148].

High pressure can at times improve the results of a few MCRs, in particular when domino-[4 + 2] or domino-[3 + 2] cyclic additions follow [149].

Various forms of the energy can be transmitted by excitation of certain bonds such as carbonyl or hydroxy and amino functional groups via: microwaves (MW) [38,150,151,152,153], infrared (IR) [154] and ultrasound (US) [38,155]. They can cause rather substantial effects on the MCRs. In this regard, there are some astonishing examples in connection to thermal energy [38]. 

Green concepts, based on rational considerations, are increasingly included in MCR chemistry [124,133,156,157,158]. There is a renaissance of catalyst-free synthesis [124].

All these criteria provide very good conditions for saving resources and energy, as well as environmentally-safe and friendly technology with regards to the environment and process and safety engineering. The atom balance is usually ideal. The key criterion in practice, synthesis efficiency, is calculated and evaluated in the following appropriate examples.

### 9.2. Several Natural Products and Drugs Syntheses via MCRs, Their Features and Comparisons

Some syntheses of natural products should contribute as examples to understanding the simplification of chemical synthesis. Efficiencies are calculated following the method in Section 8 and Section 10, the features of some individual syntheses are discussed and amazing initiatives to finding solutions for the concerted simplification of the synthesis chemistry are proposed. First, we will look at examples with a strong reduction of the number of steps.

#### 9.2.1. Nifedipine and Xylocain Syntheses

**Scheme 11:**
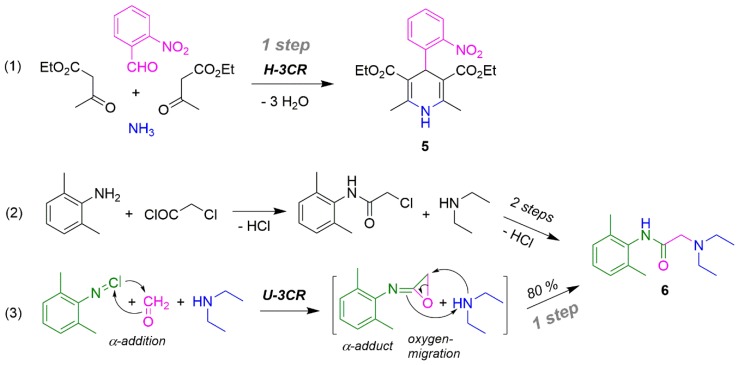
Simple drugs syntheses (1) and (3) via MCR and analogous reactions (2).

In the ideal case, synthesis chemistry should produce a compound in one step. With nifedipine **5** this succeeded via Hantzsch-3CR (Scheme 11, Reaction (1)) [159]. An efficiency >50% is now possible for the first time. In the synthesis of xylocaine (**6**) the conventional two-step synthesis (2) via Ugi-3CR is reduced to one step (Reaction (3)); with half the number of steps, the yield of **6** is 80% [101].

#### 9.2.2. Shortened Crixivan Synthesis

**Scheme 12:**
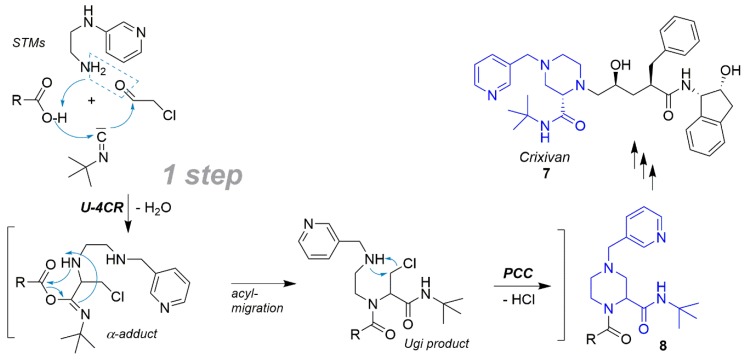
Important piperazine pharmacophore **8** for shortened Crixivan synthesis.

Another example for a strong reduction of steps using an MCR is present in the synthesis of the HIV protease inhibitor crixivan. Using Ugi-4CR the important piperazine pharmacophore **8** could be used as a component in the large scale synthesis of crixivan (**7**) by the Merck Company and so the number of steps was reduced by six steps from 18 down to 12 (33%) (Scheme 12) [160,161].

In both the following syntheses in Section 9.2.3 and Section 9.2.4, the focus is on the number of steps *and* yield of the synthesis with rational efficiency calculation.

#### 9.2.3. Eicteinascidin 743 Total Synthesis

**Scheme 13:**
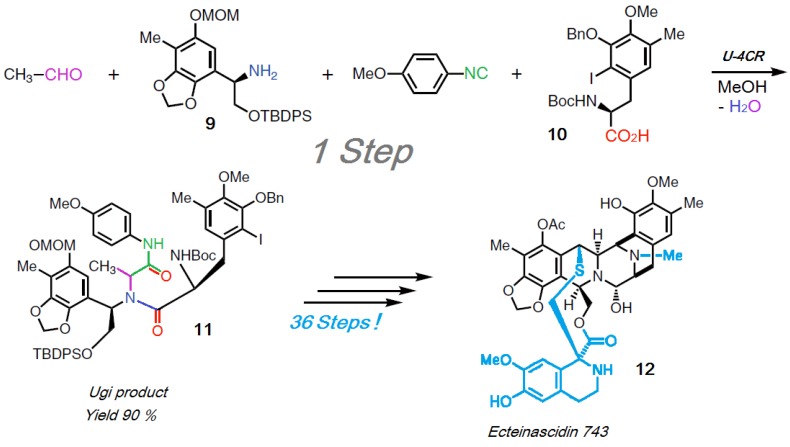
Total synthesis of eicteinascidin 743.

The key step of the synthesis of the natural compound ecteinascidin-743 (**12**) is a U-4CR based on Scheme 13 [162], delivering the Ugi product **11** with a 90% yield.

2/3 (24 C-atoms) and a greater part of the scaffold of the target product **12** (36 C-atoms) are generated in one step, presented together with the concentrated synthesis capacity of this U-4CR. For the rest of the synthesis another 36 traditional steps (!) are needed. Luckily, all synthesis data were complete and available in the publication [162] so the efficiency assessment based on this was exact.

The complete synthesis of **12** via 45 steps gives an overall yield of y_oa_ = 0.076% and an efficiency of Eff_Synth_ = 0.0017%. That is extremely low, but the completely distinct distribution of the numbers on the synthesis sections is more interesting: while it brings the synthesis of **9** and **10** and their coupling by means of U-4CR to **11** (and so 2/3 of the TM **12**) with nine and eight steps at y_oa_ = 44% and Eff_Synth_ = 5%, the values practically dwindle in the following 36 steps towards **12**, y_oa_ = 1.9%, Eff_Synth_ = 0.053%. One recognises very clearly the steep decline caused by the outright destructive influence of the high number of steps. It would be urgently necessary to consider whether and how the number of synthesis steps could be extremely reduced using higher order MCRs [163] and in particular their combined applications [164] and tools, such as multi-catalysis (Section 5.3).

#### 9.2.4. Smart Eurystatin Synthesis

**Scheme 14:**
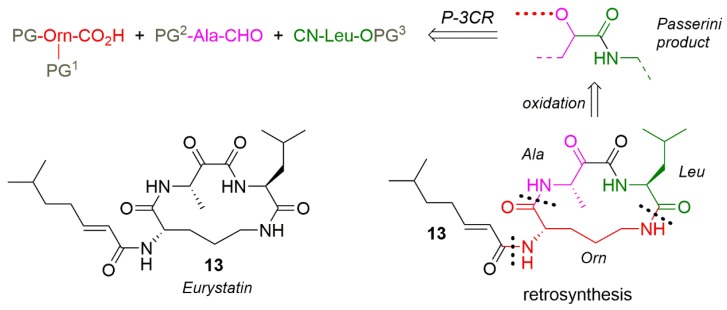
Retrosynthesis of eurystatin, indication for P-3CR.

This synthesis of natural product eurystatin (**13**) [165] is well matured and reasonably short thanks to intelligent chemical reactions considerations. Except for an additional carbonyl function group there is a cyclopeptide-derivative from alanine, leucine and ornithine, derivatized with 2-isoocteneoic acid. The retrosynthesis already indicates a P-3CR being the precursor molecule in the synthesis (Scheme 14).

**Scheme 15:**
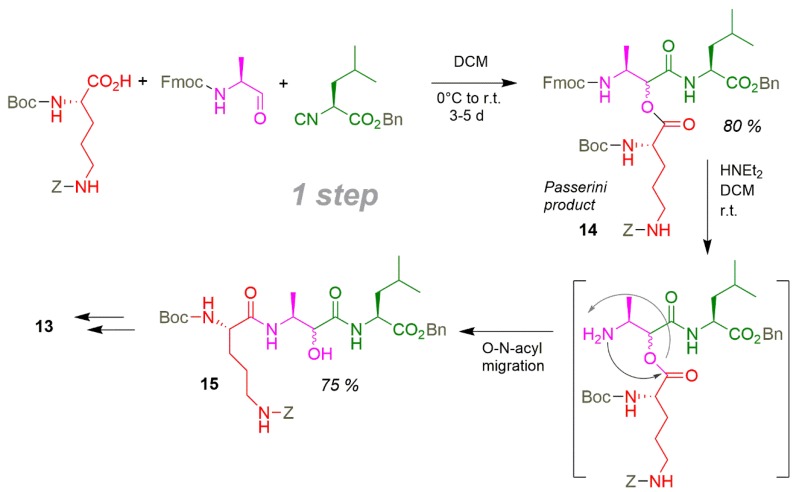
Smart P-3CR/deprotection/acyl-migration sequence in the synthesis of eurystatin.

The smart application of the thermodynamically driven *O*-*N*-acyl migration of the ornithin derivative from the hydroxyl group in the Passerini product **14** to the vicinal amino group of the alaninol to **15** saves several conventional synthesis steps (Scheme 15). Cyclisation of **15** by coupling after cleavage of Bn- and Z-residues succeed directly the ring closure of the eurystatin scaffold. The efficiency balance can be presented as: overall yield y_oa_ = 29%, eight steps, synthesis efficiency Eff_Synth_ = 3.6% and average yield y_av_ = 86%.

As the greater part of the synthesis consists of a classical protective groups based peptide synthesis, application of multi-functional catalysis from Section 5.3 (Scheme 4) could result in the addition deletion of three steps down to five.

#### 9.2.5. Tandem-U-4CR/IMDA/ROM-RCM Synthesis

**Scheme 16:**
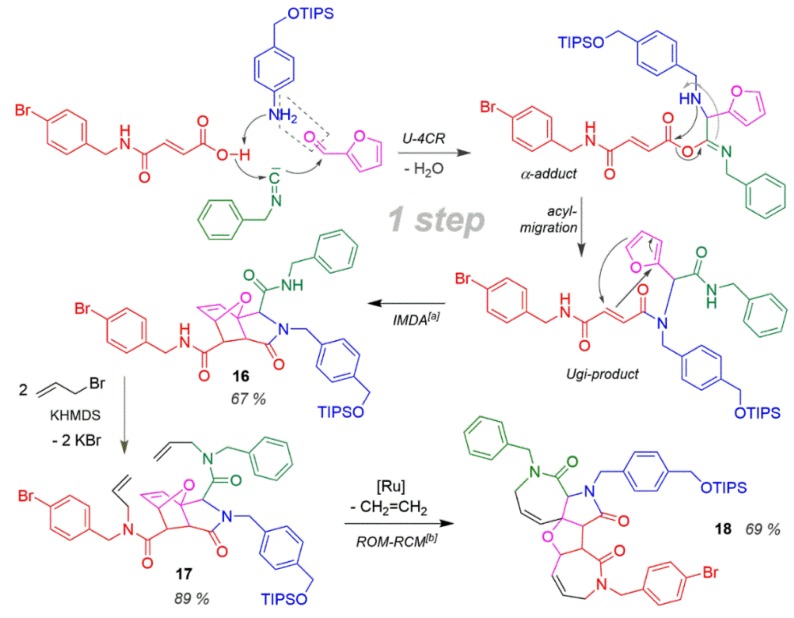
Tandem-U-4CR/IMDA/ROM-RCM synthesis. [a] Intramolecular Diels-Alder reaction. [b] Ring opening metathesis—ring closure metathesis.

This synthesis is an example of highest diversity and complexity of the target molecule, which through suitable combination (Scheme 16) of a U-4CR, Domino-Diels-Alder and a ROM-RCM reaction in three steps via the products **16** and **17** generated two annulated azepinone, furan and pyrrole heterocyclic compounds in product **18** [166] and thus delivered the starting approach for a reliable concerted simplification methodology of the synthesis chemistry. The efficiency data, y_oa_ = 41%, Eff_Synth_ = 14%, are very good with regards to the outstanding structural result.

#### 9.2.6. Multi-Function-Component Reactions (MFCRs) [H,I]

The intrinsic extension of MCRs leads to an increase of functional groups of the MCR and hence an increase in functional density. When auto-balancing functional groups such as amino and carboxylic functional groups in amino acids (AA) are concerned, the respective functional group for a reaction must first be released or activated [48,167].

**Scheme 17:**
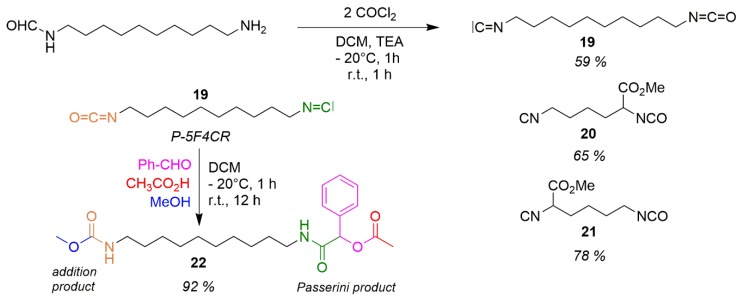
Multi-function-component reactions (MFCRs) [39].

This is different from the orthogonal highly active functional isocyanate group (strong electrophile) and isocyanide (strong nucleophile) which within one molecule I-I (isocyanato-isocyanide) are both looking for suitable partners for the MFCR (multifunctional component reaction). For the nomenclature of the MFCR refer to [38,39]. Scheme 17 introduces the I-I **19**, which is produced from formamido-amine by means of phosgene (Scheme 5) [H]. Syntheses of Lysin-derivatives **20** und **21** succeed in the same manner. **19** reacts in an additions/Passerini reaction P-5F4CR and delivers the additions/Passerini product **22** with a 92% yield [39,168]. The increase of density of functions is accompanied by a grave decrease of synthesis steps compared with a sequential synthesis [I].

#### 9.2.7. Telaprevir, Combined MCRs for a 50% Shorter Synthesis

**Scheme 18:**
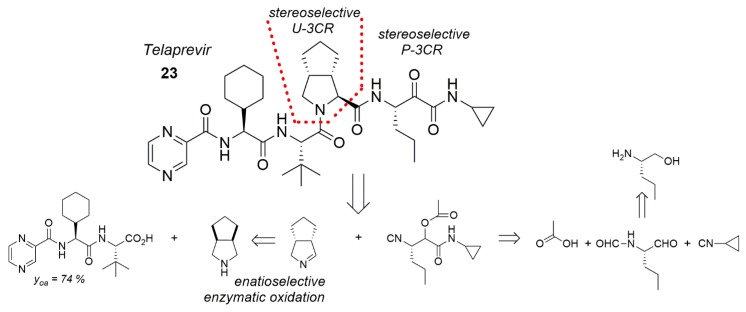
Telaprevir synthesis by combined U-3CR/P-3CR/enzymatic catalysis.

The HCV NS3 protease inhibitor telaprevir (**23**) used against hepatitis C could be synthesized in less steps by combining a Ugi-3CR, Passerini-3CR and a biocatalysis (Scheme 18) [169]. Thus, the synthesis route could be shortened by more than 50% vs. hitherto predominantly peptide-chemical syntheses [170]. Particularly the “right” section of **23** could be led by stereoselective U-3CR and stereoselective P-3CR as well as enantioselective enzymatic oxidation on the shorter road to success.

#### 9.2.8. One-step Syntheses of Oxazepinones from Carbohydrates and α-Amino Acids

**Scheme 19:**
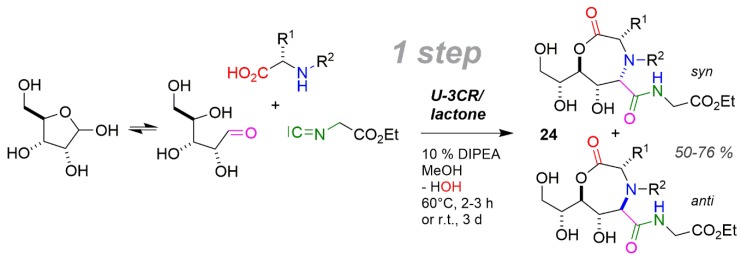
Carbohydrate carbonyl in U-3CR/lactonisation one-step synthesis of oxazepinones.

The strong dependence of carbohydrate chemistry on protective groups is well known [171], but MCRs like Ugi reactions are insensitive to hydroxy groups and thus appropriate for application with carbohydrates as carbonyl component to save material synthesis steps. This has been achieved in a one-step U-3CR/lactonisation of D-ribose with α-amino acids (AA) and ethyl isocyanoacetate (Scheme 19) [76,172], furnishing oxazepinones 24 in yields of around 50% up to 76%. The *syn/anti* values of 24 are 67/33 to 91/9. For the mechanism of the U-3CR see Scheme 11 Reaction (3). The reward of this high-performance synthesis is an exceptionally high efficiency of up to 76%.

#### 9.2.9. Mild Esterification by P-3CR

**Scheme 20:**
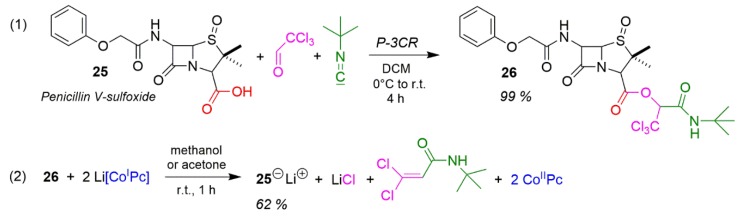
Application of P-3CR for esterification reactions.

The presentation of the diversity of methodologies in the MCR domain is rounded off by the application of the Passerini reaction which can solve so many practical problems, notably the esterification under extremely mild conditions according to Scheme 20 Reaction (1) [13,173]. This reaction enables simple and high-yield esterification of readily decomposing β-Lactam antibiotics with excellent yields. The reaction between penicillin V-sulfoxide, chloral and *tert*-butyl isocyanide produces the penicillin-V-sulfoxide 1′-*tert*-butylaminocarbonyl-2′,2′,2′-trichlorethylester **26** as colourless crystals with a yield of 99%. Cleaving off the alkyl residue (Reaction (2)) furnishes **25** with a yield of 62% [13]. The mechanism of removal works according to Scheme 1.

## 10. Synthesis-Efficiency of MCRs

**Figure 6:**
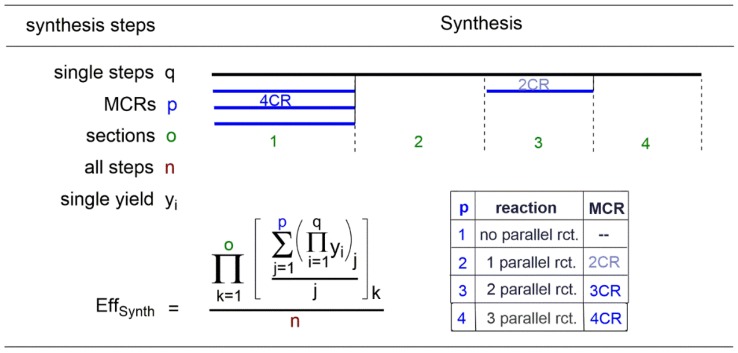
Algorithm “Sythesis efficiencies including MCRs” phrased as mathematical equation.

Naturally, all calculations in Section 8 apply fully to the MCR syntheses. Parallel reactions originating from the synthesis of the precursors of the components **C** > 1 can be added. One 3CR can therefore have up to two, and one 4CR up to three, parallel reactions in their precursor syntheses. As the parallel reactions are not sequential, they do not have a multiplier character regarding yields and efficiency, but must be suitably included in the overall calculation, which is a summation. The partial sections (of the parallel reactions) are incorporated into the algorithm as weight-averaged yields of the arithmetic mean values of the parallel reactions. The number of steps is simply the sum of all steps in the respective section. The algorithm can be expressed as a mathematical equation for the synthesis efficiency as shown in Figure 6.

The quantitative synthesis efficiency Eff_Synth_ specifically refers only to the concrete criteria of yield and number of steps, and is therefore particularly reliable and independent of other “soft” evaluation criteria that can complement this value. Regarding the reaction work-up, MCR products can mostly be easily isolated and purified. The lack of akin products could be explained by MCRs nature as formal addition reactions and their “well-tempered” reaction enthalpies. The latter are the result of a balanced system of several reaction-parts acting with each other (see Section 9.1).

Evaluating syntheses in Section 9.2.1, Section 9.2.2, Section 9.2.3, Section 9.2.4, Section 9.2.5, Section 9.2.6, Section 9.2.7, Section 9.2.8 and Section 9.2.9 in cumulo with regards to their over-all and embedded MCRs efficiencies and in comparison with each other, it is significantly evident, that MCRs are much more able than 2CRs, to generate main parts of complex products, as demonstrated in Section 9.2.2, Section 9.2.3, Section 9.2.4, Section 9.2.5 and Section 9.2.7. Also, complete products can be synthesized in one-step MCRs, as shown in Section 9.2.1. Even highly diverse products are easily produced from simple and low-cost STMs by a one-step MCR, as evidenced in Section 9.2.8. Another progress has happened by synchronously running of two orthogonal reactions at the same molecule, presented in Section 9.2.6.

The requirements of *Green Chemistry* are fulfilled, such as excellent atom-efficiency, the avoidance of waste, better performing compounds, eco-compatible solvents, renewable materials, and energy reduction.

## 11. Multicomponent Chemistry—The Future

Section 9 presents the diversity of prospects for MCR chemistry in a systematic (Scheme 9 and Scheme 10) and a perspective manner using illustrative examples (Scheme 11, Scheme 12, Scheme 13, Scheme 14, Scheme 15, Scheme 16, Scheme 17, Scheme 18, Scheme 19 and Scheme 20 The multitude of reactions and their combinations with each other and within biology opens up the exponentially growing scope of reactions for this methodology [36,109,113,174].

If one links the exponential growth of potential MCRs with their intrinsic exponentially increasing numbers of products, one can quickly discern the unique potential of this methodology which enables the *production and synthesis at will of molecules of whatever structure and quantity*. Syntheses via the MCR methodology allow us to achieve what Nature has already been doing for a very long time in its tried and tested manner. In comparison, the possibly virtually unlimited number of products will be attained relatively fast. Part 2 of the chemical evolution (Figure 2) lies ahead and this time humans are involved. How do we achieve the afore mentioned opportunities and goals?

1. Creation of further MCRs and MFCRs that can create numerous structural elements of chemical compounds and show high diversity.
2. Creation of further MCRs of a higher order (with as many components as possible) that will exponentially increase the variability and the quantity of products.
3. Creation of further MFCRs with as many functional groups as possible that will strongly increase the complexity of products in particular.
4. Combining MCRs with each other in a rational way and number thus making it feasible to extremely decrease the number of synthesis steps.
5. Creating an algorithm in order to verify procedures 1 through 4.
6. New tandem, domino and cascade reactions to extend the MCRs or their combinations, which will further strongly decrease the number of synthesis steps. This will result in double the reduction in steps in conjunction with the MCR combinations.
7. Process development of one-reactor synthesis as a tool to reduce the number of synthesis steps.
8. Multifunctional catalysts and multi-catalysis cascades (MCC) as tools to reduce the number of synthesis steps.
9. High pressure as tool for increasing scope and yield of certain MCRs.
10. Identifying and developing new tools to simplify production.
11. Rational and practice-based efficiency calculation in the prognosis of planned and the analysis of syntheses performed with a feed-back effect on synthesis optimisation.

In order to support the aforementioned concept, important known data will be determined:

12. Databases (e.g., SciFinder) with information about the target products and intermediates of the synthesis plan.
13. Databases (e.g., SciFinder) about the applications of the planned MCRs and their scope and limitations. Synthesis data and synthesis protocols, references and details.
14. Drug Design & Discovery.
15. Specialist literature for understanding MCRs and their mechanisms, including probable mechanisms, in order to assess undesired side reactions.

*With respect to 1:* New and in particular novel MCRs [38,45,61,81,82,112,113,154,157,174,175,176,177,178] are the basis and guarantee for high diversity and therewith the capacity to produce many structurally-different compounds. A method has been found to design MCRs going from libraries of compounds to libraries of reactions [176].

*With respect to 2:* In order to obtain very high product quantities using the MCRs (libraries), MCRs of a higher order with component numbers >4 are particularly suitable [163], such as 5CR [179], 7CR [180] and 8CR [181]. Table 5 shows the relationship between exponential components and product numbers.

*With respect to 3:* As much as possible high complexity can be obtained by as many as possible functional groups on the components of the MCR. Just one additional functional group already causes a new dimension in the scope of reactions [39,112,113,174,182]. Further to the functional density, one has to look at the reactivity. While passive “pairs” such as amino and carboxylic groups (AAs) first have to be activated, active “pairs” such as isocyanate-isocyanide are able to enter 2 different MCRs or reactions in a highly selective way (Scheme 17) [39].

*With respect to 4:* The well-planned combination of MCRs (unions) [170,182,183,184,185] opens up a further method for reducing the number of synthesis steps with simultaneous structural assembly based on a modular building concept, such as Scheme 18.

*With respect to 5:* The relative reactivities of the functional groups of the MCRs are important criteria for an algorithm which is useful when looking for new MCRs including computer-aided methods [186]. Unexpected products lead to new MCRs. Algorithm-based methods for the discovery of novel multicomponent reactions [187,188].

*With respect to 6:* During MCR development, suitable domino reactions are also designed, creating indole and steroid alkaloids [122]. Functional π-systems [189], norbornene [190] and A3-coupling [115] with MCRs/domino reactions were deployed for DOS. Proline-catalysed domino reactions are used for the synthesis of heterocyclic groups using MCRs [117]. MCR/domino processes develop through rational design and serendipity [114]. 

*With respect to 7:* The advantages of MCR one-pot processes are demonstrated in several examples of the production of pharmaceutical products [164,191].

*With respect to 7 + 8:* Suitable multifunctional catalysis can greatly contribute to make low-step syntheses using MCR. This has been performed in one-pot sequential reactions [164]. 

Scheme 4 shows another convincing example. The process reduces a classical synthesis sequence of the peptide chemistry from three steps down to one! The efficiency of the synthesis is three times higher than the 3-step synthesis. This is achieved using the switchable catalyst palladium-phthalocyanin. The method chosen can be generally and widely used for the synthesis of peptides, and can be repeated successively in a single reactor if necessary. 

*With respect to 9:* Some MCRs could only be performed when high pressure catalysis was applied [149].

*With respect to 11*: Three multicomponent reactions, among them the Strecker reaction (without any catalyst), have proven to be very efficient in the generation of a diversity of polyfunctionalized molecules [192]. A review provides an overview of the exploitation of multicomponent reactions for the synthesis of nonsteroidal anti-inflammatory drugs: Multicomponent reactions are more efficient, cost effective and economical than traditional methods [193,194].

In Section 8 and Section 10 an algorithm has been developed to calculate synthesis efficiency based on hard criteria overall yield and steps of a synthesis. The algorithm also takes parallel reactions and MCRs and delivers an exact result of the efficiency even on comprehensive and complex syntheses. 

*With respect to 14:* Many capabilities and properties of MCRs make them particularly suitable for Drug Design and Discovery [91,177,188,195,196] primarily with the higher order MCRs, which can easily generate very large libraries as Table 5 clearly indicates. The availability of MCRs has become so diverse that, in combination with each other and with post condensation modifications such as tandem, cascade or domino reactions, we can now look at an extremely high structural diversity of products.

## 12. Conclusions

**Figure 7:**
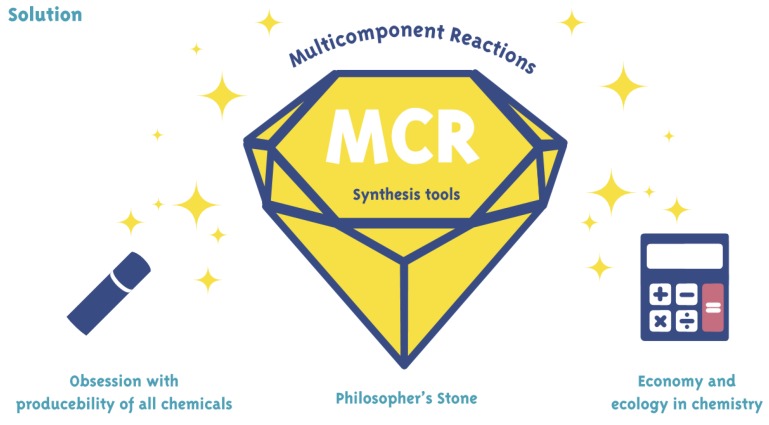
Solution of the discrepancy in Section Prologue in performing syntheses by concerted simplification in an elegant and highly efficient way.

Research results relating to the aforementioned points are piling up in the literature. The time has now come to coordinate these highly valuable individual results from the most diverse domains of organic (Section 3, Section 4, Section 5, Section 6, Section 7 and Section 8) and MCR chemistry (Section 9, Section 10 and Section 11) in accordance with the above-mentioned 15 points-scheme, to bundle the already strong individual effects and to concentrate on the actual synthesis. Under the fulfilment of *Green Chemistry* requirements (Section 10) the concerted *simplification of the chemical/biological synthesis* (Figure 7) will subsequently take great strides, providing mankind, science and the economy with tailor-made and keenly low-cost materials, drugs and chemistry libraries.

## 13. Biographical Data of Heiner Eckert

A. During my search for a thesis in the Faculty of Chemistry at the Technical University of Munich, the newly appointed Professor Ivar Ugi, who wished to be on first-name basis, immediately offered me several topics for selection, all based around his 4CC method, the Ugi four-component condensation. A novel protective group technique needed to be developed and integrated within the 4CC-based synthesis of peptides. Ugi had just brought this idea with him from California where he was still supervising several PhD theses at his former base, the University of Southern California. While there, he met his old friend Gerhard Schrauzer (Professor at the University of California) who was researching the synthetic function-analogous complexes of vitamin B_12_, the cobaloximes. As this interdisciplinary project fascinated me as a “greenhorn”, I enthusiastically accepted, not realising what it meant to gain a foothold as the sole participant in this project—a valuable experience!

B. I had the idea, to employ the dye-stuff cobalt-phthalocyanine as “technical vitamin B_12_” into the project, and this opened self-reliant work for me, which was strongly supported by Ugi.

C. The ability of the new protecting groups technology which I developed to produce highly sensitive compounds such as β-lactam antibiotics (Figure 3) made me very proud. These particular characteristics of the reduced metal-phthalocyanines, and their exceptional chemically reactive features, drew the interest of Bayer AG, who have since, together with Ugi and myself, registered various patents, both in the area of pigment production [14] and in the field of chemical semi-synthesis of β-lactam antibiotics [15,16]. The respective work at Bayer was stopped after the biochemical synthesis processes were established. This decision taken by the global player already shows the limits of practicability of classic chemical multi-stage synthesis. Meanwhile I achieved my doctorate as “Dr. rer. nat.” with the grade “summa cum laude” with Ugi.

D. Up to this day I consider the switchable specificity of the catalyst PdPc between three partly orthogonal catalyst patterns with their multiple application patterns as a special highlight of my work in this domain. It constitutes the basis of the “Eckert Hydrogenation Catalysts” [20].

E. One of the first preparations on the agenda at the start of my thesis work was the production of several 100 g of a chlorocarbonic acid ester from phosgene and an alcohol. Concern was manifest and initial considerations for a substitute began to arise. But it took another 10 years before an opportunity appeared. During the production of diphosgene, a substitute for phosgene that had just arrived on the market, a small amount of white solid remained following distillation and I decided to analyze it instead of discarding it.

F. These results encouraged me to sell triphosgene as a commercial phosgene substitute via my newly founded company Dr. Eckert GmbH and the chemicals trade reacted very rapidly, increasingly taking over distribution. After several years, China entered production and currently provides the entire world with thousands of tons of triphosgene.

G. In order to be able to greatly extend the business activities of the company, I took an interested investor on board who shortly afterwards wanted to proceed doing business on his own. And thus, at the end of the millennium, I sold the company for a share in its profits to the investor.

H. The very first and successful production of isocyanato-isocyanide really was the most exciting experience of my work history in chemistry. The contradictory existence of these compounds within one molecule was assumed to be impossible by many colleagues. Two such strong, but incompatible functional groups should not be able to coexist and would react with each other at very moment of formation. This made the analytical confirmation feel even sweeter.

**Figure 8:**
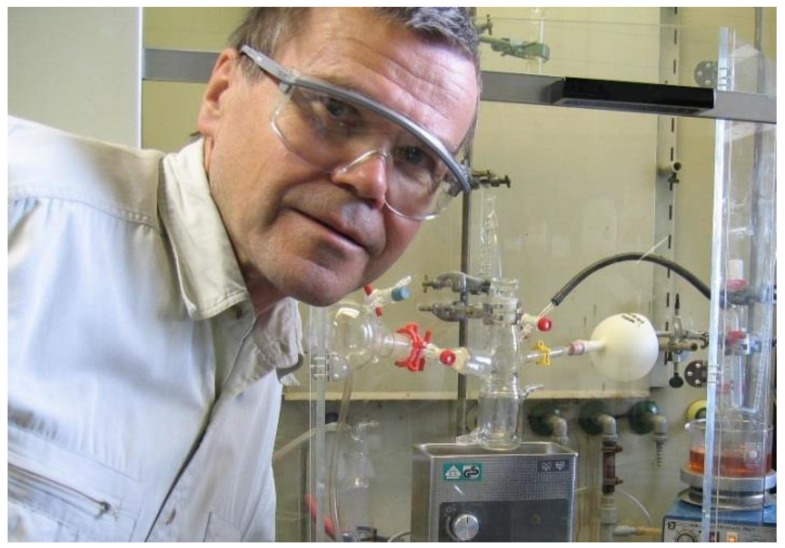
Heiner Eckert at the laboratory [J].

I. After a comprehensive and diverse research in chemistry done over three decades I had time to perform the overdue habilitation [25] achieving the “venia legendi” in chemistry at Technical University of Muenchen (TUM) at the age of 59 years (Figure 8).

J. After two years in pension I accepted the call of the biochemistry start-up-company BSAZ Biotech (Hangzhou) Ltd. in China to become its Vice President. In August 2015, I was presented with the “Quianjiang Friendship Award 2015” for foreign experts in the city of Hangzhou and the “West Lake Friendship Award 2015” of the government of Zheijang Province in October.

## Figures and Tables

**Table 1:**
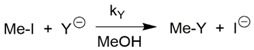
Relative nucleophilicity n_MeI_ = log (k_Y_/k_MeOH_) for the following reaction at 25 °C [5].

Nucleophile Y	n_MeI_	Structures of Supernucleophiles
MeOH	0.00	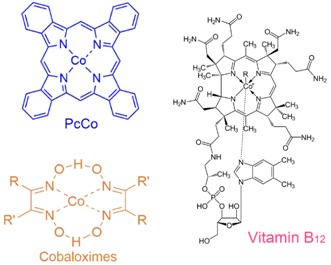
Cl^−^	4.37	
Br^−^	5.79	
CN^−^	6.70	
I^−^	7.42	
S_2_O_3_^−^	8.95	
Ph-S^−^	9.92	
[PcCo^I^]^−^	10.8	
Cobaloximes(I)	10.5–14.3	
Vitamin B_12s_	14.4	

**Table 2:** Catalysis-pattern P_i_ (i = 1–3) of the catalyst Pd^**Z**^Pc transforming A-F^1^ to A-F^2^ referred to Scheme 3. [a] Corbobenzoxy residue. [b] 2,2,2-Trichloro-*tert*-butoxycarbonyl residue. [c] Benzyl residue.

F^1^	A	z = 2 *P*_1_ pH < 9	z = 2 *P*_2_ pH > 11	z = 1 *P*_3_	F^2^ (*P*)
C=C, C=N	Alkyl, Aryl	**+**	**+**	**+**	CH-CH, CH-NH
NO_2_, CH=O	Aryl	**+**	**+**	**+**	NH_2_, CH_3_ (*P_3_*: CH_2_-OH)
NO_2_, CH=O, Hal	Alkyl	**−**	**−**	**+**	NH_2_, CH_2_-OH, H
Hal	Aryl	**−**	**+**	**−**	H
C=O, COCl, CN	Alkyl, Aryl	**−**	**−**	**+**	CH-OH, CH_2_-OH, CH_2_-NH_2_
Z, CBZ ^[a^]	NH	**+**	**+**	**−**	H
TCBOC ^[b]^, β-Haloalkoxy	NH, O	**−**	**−**	**+**	H
X-Bn ^[c]^ (X = O, N, CON)	Alkyl, Aryl	**−**	**−**	**−**	F^1^
CO_2_R, CONR_2_	Alkyl, Aryl	**−**	**−**	**−**	F^1^

**Table 3:** Physical, chemical, thermochemical, and toxicity data of phosgene and triphosgene [28,29].

Data	Phosgene (4)	Triphosgene (3)
Melting point m.p. (°C)	–118	80
Boiling point b.p. (°C)	8	206
Vapor pressure at 20 °C (Pa/mmHg)	162,000/1215	20/0.15
Relative reactivity to MeOH	170	1
ΔH (conversion of **3** to **4**) (J·g^−1^)	-	+9
ΔH (decomposition of **3**) (J·g^−1^)	-	–243
Toxicity LC_50_ (mg·m^−3^)	7	41.5
Toxicity LC_50_ (mMol·m^−3^)	0.07	0.14

**Table 4:** Overall yields y_oa_ dependent on geometric average yields y_av_ and numbers n of steps of syntheses. 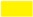 highly profitable short syntheses, 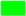 usual pharma production, 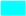 high-chem research preparations.

Number of Steps	Over-All-Yield y_oa_ (%)							
	Geometric Average Yields y_av_ (%)							
n	99	98	95	90	80	70	60	50
2	98	96	90	81	64	49	36	25
3	97	94	86	73	51	34	22	13
4	96	92	81	66	41	24	13	6.3
5	95	90	77	59	33	17	7.8	3.1
6	94	89	74	53	26	12	4.7	1.6
7	93	87	70	48	21	8.2	2.8	0.8
8	92	85	66	43	17	5.8	1.7	0.4
9	91	83	63	39	13	4.0	1.0	0.2
10	90	82	60	35	11	2.8	0.6	0.1
12	89	78	54	28	6.9	1.4	0.2	0.02
15	86	74	46	21	3.5	0.5	0.04	0.003
20	82	67	36	12	1.2	0.08	0.004	
30	74	55	21	4.0	0.1	0.002		
40	67	45	13	1.5	0.01			
50	61	36	7.7	0.5	0.001			10^−13^

**Table 5:** Calculated products as function of component **C** or function **F** numbers in MCRs.

MCR				STM-sets	
MFCR	2	4	10	30	100
2CR	4	16	100	900	10.000
P-3CR	8	64	1000	27,000	1,000,000
U-4CR	16	256	10,000	810,000	100,000,000
P-5F4CR	32	1024	100,000	24,300,000	10,000,000,000
5CR	32	1024	100,000	24,300,000	10,000,000,000
7CR	128	16,384	10,000,000	21,870,000,000	100,000,000,000,000
8CR	256	65,536	100,000,000	656,100,000,000	10,000,000,000,000,000
